# Increased expression of ACTH (*MC2R*) and androgen (*AR*) receptors in giant bilateral myelolipomas from patients with congenital adrenal hyperplasia

**DOI:** 10.1186/1472-6823-14-42

**Published:** 2014-05-12

**Authors:** Madson Q Almeida, Laura C Kaupert, Luciana P Brito, Antonio M Lerario, Beatriz M P Mariani, Marta Ribeiro, Osmar Monte, Francisco T Denes, Berenice B Mendonca, Tânia ASS Bachega

**Affiliations:** 1 Divisão de Endocrinologia e Metabologia, Laboratório de Hormônios e Genética Molecular/LIM42, Hospital das Clínicas da Faculdade de Medicina da Universidade de São Paulo, Av. Dr. Enéas de Carvalho Aguiar, 155, 2 andar, Bloco 6, São Paulo, SP 05403-900, Brasil; 2 Instituto do Câncer do Estado de São Paulo (ICESP), Faculdade de Medicina da Universidade de São Paulo, São Paulo, Brasil; 3 Unidade de Endocrinologia e Metabologia, Departamento de Clínica Médica, Faculdade de Ciências Médicas da Santa Casa de Misericórdia de São Paulo, São Paulo, Brasil; 4 Serviço de Urologia, Hospital das Clínicas, Faculdade de Medicina da Universidade de São Paulo, São Paulo, Brasil

**Keywords:** Adrenal myelolipoma, Congenital adrenal hyperplasia, ACTH, *MC2R*, Androgen receptor, Clonality analysis

## Abstract

**Background:**

Although chronic adrenocorticotropic hormone (ACTH) and androgen hyperstimulation are assumed to be involved in the pathogenesis of adrenal myelolipomas associated with poor-compliance patients with congenital adrenal hyperplasia (CAH), the expression of their receptors has not yet been demonstrated in these tumors so far.

**Methods:**

We analyzed *Melanocortin 2 receptor* (*MC2R*)*, Androgen Receptor (AR), Leptin* (*LEP*)*,* and *Steroidogenic factor 1* (*SF1*) expression using real-time qRT-PCR in two giant bilateral adrenal myelolipomas from two untreated simple virilizing CAH cases and in two sporadic adrenal myelolipomas. In addition, the X**-**chromosome inactivation pattern and CAG repeat number**s** in *AR* exon 1 gene were evaluated in the 4 cases.

**Results:**

The *MC2R* gene was overexpressed in myelolipomas from 3 out of 4 patients. *AR* overexpression was detected in 2 tumors: a giant bilateral myelolipoma in a CAH patient and a sporadic case. Simultaneous overexpression of *AR* and *MC2R* genes was found in two of the cases. Interestingly, the bilateral giant myelolipoma associated with CAH that had high androgen and ACTH levels but lacked *MC2R* and *AR* overexpression presented a significantly shorter *AR* allele compared with other tumors. In addition, X-chromosome inactivation pattern analysis showed a polyclonal origin in all tumors, suggesting a stimulatory effect as the trigger for tumor development.

**Conclusion:**

These findings are the first evidence for *MC2R* or *AR* overexpression in giant bilateral myelolipomas from poor-compliance CAH patients.

## Background

Adrenal myelolipomas are benign non-functioning tumors composed of adipose tissue and hematopoietic elements resembling bone marrow [1]. These tumors account for up to 8% of adrenal incidentalomas [2]. Adrenal myelolipomas are usually asymptomatic but can cause compressive symptoms. Typically, they have a fat signal intensity on T_1_-weighted magnetic resonance (RM) [2]. Interestingly, myelolipomas have been described in the setting of adrenocorticotropic hormone (ACTH) excess, such as classical congenital adrenal hyperplasia (CAH) [1], Cushing disease [3] and Nelson syndrome [4]. Recently, Nermoen et al. [5] reported a 4% frequency of adrenal myelolipomas (4 out of 101; 3 of them with bilateral myelolipomas) in a large group of unselected patients with 21-hydroxylase deficiency (21OH). Adrenal myelolipomas can rarely present as giant bilateral masses, but approximately 14 cases of giant bilateral myelolipomas have been described in association with CAH [1,5-9].

Several mechanisms have been proposed to explain the pathogenesis of adrenal myelolipomas, such as embryonic bone marrow rests in adrenal tissue, adrenal embolization of bone marrow cells and metaplasia of adrenocortical cells [10,11]. Although chronic ACTH hyperstimulation is thought to be involved in the pathogenesis of adrenal myelolipomas based primarily on the finding of bilateral tumors in poor-compliance CAH patients, this hypothesis remains to be confirmed. *Melanocortin 2 receptor (MC2R)* is selectively activated by ACTH and encodes a G-protein coupled receptor. Indeed, *MC2R* and *androgen receptor* (*AR*) expression was previously evaluated in a single case of giant bilateral myelolipoma in a CAH patient and was negative using a semi-quantitative approach [1]. However, considering the high frequency of association between giant bilateral myelolipomas and CAH, we hypothesized that *ACTH* and *AR* might have a role in the pathogenesis of myelolipomas.

In this study, we analyzed *MC2R* and *AR* expression as well as nCAG *AR* repeat numbers in two bilateral giant myelolipomas from CAH patients and two unilateral sporadic myelolipomas. Additionally, clonality was evaluated through X-chromosome inactivation analysis. Our data indicated that *MC2R* and/or *AR* were involved in the pathogenesis of myelolipomas associated with CAH, suggesting a stimulatory hormonal effect as a trigger for tumor growth. These findings are the first evidence for ACTH and androgen roles in giant bilateral myelolipomas in CAH patients and sporadic cases.

## Methods

The study was approved by the Ethics Committee of Hospital das Clínicas, São Paulo University and from Santa Casa de Misericordia Hospital, and informed written consent was obtained from all patients for participate in the study and for the publication of data and/or images. Blood and tissue samples were collected from the patients after informed consent was obtained. Four patients with myelolipomas were evaluated in this study: two giant bilateral adrenal myelolipomas from two untreated simple virilizing CAH cases and two sporadic adrenal myelolipomas. Abdominal masses were identified with computed tomography (CT) or magnetic resonance imaging (MRI), and pathology confirmed myelolipoma after bilateral or unilateral adrenalectomy.

### Quantitative real-time PCR

After surgical resection, tumor fragments were immediately frozen in liquid nitrogen and stored at -80°C until total RNA extraction using Trizol reagent (Invitrogen, Carlsbad, CA). cDNA was generated using a High Capacity kit (Applied Biosystems, Foster City, CA, USA). Quantitative real-time PCR (qRT-PCR) was performed with an ABI Prism 7700 sequence detector using TaqMan gene expression assays (Applied Biosystems, Foster City, CA). The assay identifications were the following: *MC2R* (Hs00265039_s1), *AR* (Hs00907244_m1), *SF1* (Hs00610436_m1) and *LEP* (Hs01084494_m1). *Beta-actin* (*ACTB*) and *Glyceraldehyde-3-phosphate dehydrogenase* (*GAPDH*) genes were used as endogenous genes. A commercial pool of adipose tissue and adrenal tissue was used for comparisons (CLONTECH, BioChain, and Ambion). Relative quantification was performed using the 2^-ΔΔCT^ method [12]. Overexpression was defined as a two-fold change in comparison to normal adipose tissue.

### X-Chromosome inactivation and CAG repeat numbers of the *AR* gene

Genomic DNA was obtained from tumors using standard procedures. The X-chromosome inactivation pattern and CAG repeat numbers were determined as previously described [13]. Briefly, PCR amplification of the CAG repeat region of *HpaII-*digested and undigested samples was carried out using primers flanking the region of interest: 5’-GCTGTGAAGGTTGCTGTTCCTC-3’ and 5'-HEX-GTGCGCGAAGTGATCCAGAA-3’. All samples were separately amplified in 50 μL reactions containing 1x PCR reaction buffer with 1.5 mM of MgCl_2_, 200 μM of deoxynucleotides, 15 pmol of each primer and 1 U of Taq DNA Polymerase (Amersham-Pharmacia, Uppsala, Sweden). Amplifications were performed under the following conditions: initial denaturation at 97°C for 5 min; amplification for 35 cycles with denaturation at 97°C for 1 min, annealing at 54°C for 45 s and extension at 72°C for 45 s; and one final extension at 72°C for 30 min. Two and 4 μL of PCR products from undigested and digested samples, respectively, were submitted to capillary electrophoresis on ABI PRISM 310 Genetic Analyzer (Applied Biosystems, Foster City, CA, USA) and analyzed by GeneScan software to determine the sizes of the amplified fragments, which were established through comparison**s** with a size marker and a sample with a known CAG repeat number in the same run. These sizes were correlated with CAG repeat numbers, as previously shown in our lab [14]. Digested and undigested samples were also assayed in the same run, and the peak height of each allele was used to determine the X-chromosome inactivation pattern.

## Results

### Clinical data

**Figure 1:**
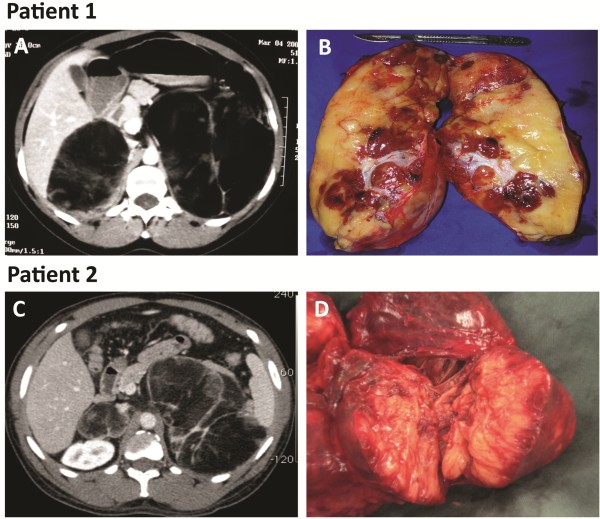
**Imaging and histopathology of giant bilateral myelolipomas in patients with congenital adrenal hyperplasia.** Patient 1: **A,** CT scan showing heterogeneous bilateral myelolipomas (left, 14 × 14 × 10 cm; right 8.9 × 8.3 × 8.0 cm). **B,** Macroscopic aspect of left adrenal myelolipomas. Patient 2: **C,** CT scan revealing large bilateral myelolipomas (left, 16 × 13 × 9.0 cm; right, 5.3x 4.3x 6.9 cm). **D,** Macroscopic appearance of left adrenal myelolipomas.

**Patient 1** was a 35-yr-old woman who presented with the simple virilizing form of CAH [p.E351V and exon 6 cluster (p.I236N, p.V237E, and p.M239K) mutations in a compound heterozygote state in the *CYP21A2* gene]. CT scan revealed giant bilateral adrenal myelolipomas (left, 14 × 14 × 10 cm; right 8.9 × 8.3 × 8.0 cm) with fat component density. She did not present to clinical follow-up and had not received any medications in the last 15 yr. Hormonal evaluation revealed extremely increased levels: basal 17OH-progesterone (17-OHP) 192 ng/mL, ACTH 1,172 pg/mL, total testosterone 949 ng/dL and androstenedione 17 ng/mL (Figures 1A and 1B).

**Patient 2** was a 52-yr-old woman who presented with the simple virilizing form of CAH (IVS2-13A/C > G/ p.I172N). CT scan revealed giant bilateral adrenal myelolipoma (left, 16 × 13 × 9.0 cm; right, 5.3 × 4.3 × 6.9 cm) with fat component density (Figures 1C and 1D). This patient had never been previously treated, and hormonal evaluation also revealed increased serum levels: 17-OHP 120 ng/mL, total testosterone 720 ng/dL and androstenedione 39 ng/mL. ACTH measurement was not available. Both CAH patients were severely virilized during adolescence and changed to male social sex. They sought medical assistance due to abdominal pain.

**Patient 3 (a 48-yr-old female) and patient 4 (a 40-yr-old female)** presented with incidental findings of sporadic unilateral adrenal myelolipoma ranging from 8 to 10 cm in the major diameter. MRI demonstrated fat tissue signal in both masses. The patients did not present any clinical presentation of hyperandrogenism and non-classical CAH was rule-out by clinical evaluation.

Briefly, the histopathological analysis of all tumors showed lobes of mature adipose tissue mixed with abundant hematopoietic tissue consisting of all three hematopoietic elements with mature and precursor cells. There were islets of cells from the zones of the adrenal cortex in the periphery of tumors as well as between hematopoietic and lipoid cells.

### Expression study

The *MC2R* gene was overexpressed in the myelolipomas of 3 out of 4 patients (Table 1). *MC2R* expression was correlated with *SF1* mRNA levels in the same tumors. Only the myelolipoma diagnosed in patient 1 did not show *MC2R* overexpression. As expected, *MC2R* mRNA levels were higher in the adrenal pool.

**Table 1:** **nCAG repeat numbers and *AR, MC2R, LEP *** **and *SF1 *** **gene expression in myelolipomas**

**Patients**	**nCAG**	AR*	MC2R*	LEP*	SF1*
1	15/21	0.1	0.6	0.2	1.0
2	20/23	8.6	853	2.1	365
3	24/25	0.1	99	0.04	6.3
4	22/30	5.0	3214	6.1	259
Adrenal pool		0.03	481	0	2462

*An adipose tissue pool was used as the reference sample in the expression analysis.

The *AR* gene was overexpressed in myelolipomas from patients 2 and 4. Low *AR* expression levels were found in the other cases (patients 1 and 3). *AR* expression was correlated with *LEP* expression in all tumors.

### Clonality and nCAG repeat number analysis

X-chromosome inactivation pattern analysis revealed a polyclonal origin in all tumors. In addition, all tumors were informative, and nCAG repeat numbers varied from 20 to 30, except in the tumor from patient 1, which had a short allele (15 repeats) (Table 1).

## Discussion

The pathogenesis of adrenal myelolipomas is unclear, but several mechanisms have been proposed to underlie the etiology. One of the hypothesis is that hematopoietic and fat elements could derive from common progenitor cells secondary to stimulatory factors [15]. In this study, we demonstrated that the *MC2R* gene was overexpressed in 3 out of 4 myelolipomas. Among these 3 cases, 1 giant bilateral myelolipoma was associated with CAH, and two patients presented sporadic myelolipomas. Chronic ACTH hyperstimulation has been proposed as the main hypothesis to explain the higher frequency of giant and bilateral myelolipomas in poor-compliance CAH patients, but it has never been previously demonstrated. Lack of *MC2R* gene expression was demonstrated in a giant adrenal myelolipoma associated with CAH employing a semi-quantitative approach to analyze mRNA expression [1]. Therefore, to our knowledge, our finding is the first evidence of *MC2R* overexpression in myelolipomas. *MC2R* overexpression was found in all but one case. Despite of chronic ACTH hyperstimulation, myelolipoma from patient 1 did not present *MC2R* overexpression.

Androgen receptors have also been implicated in the pathogenesis of myelolipomas associated with poorly controlled CAH patients [1]. In the current study, *AR* overexpression was detected in 2 tumors: a giant bilateral myelolipoma in a CAH patient and a sporadic case. *AR* expression was previously assessed in a single case of bilateral myelolipoma associated with CAH using a semi-quantitative technique, but the results were negative [1]. Here, we employed a more sensitive approach to study *AR* and *MC2R* expression. The overexpression of *AR* and *MC2R* genes was concomitantly found in two of the cases.

It has been postulated that androgens, through their interaction with androgen receptors, may play an important role in the development of specific tumors, such as ovarian and prostate cancer [16,17]. Exon 1 of the *AR* gene contains highly polymorphic trinucleotide repeats, and the length of the nCAG repeat segment is inversely correlated with the transactivation function of the *AR* gene [18]. Interestingly, the case of bilateral giant myelolipoma associated with CAH that lacked *MC2R* and *AR* overexpression had a shorter *AR* allele compared with the other tumors, suggesting that this *AR* genotype in the context of very high androgen levels may play a causative role in the development of myelolipomas. The stimulatory effect of *MC2R* and *AR* overexpression or increased *AR* transactivation activity in the development of adrenal myelolipomas could be reinforced by the finding of a polyclonal origin in all tumors described here.

## Conclusion

In conclusion, we first demonstrated here *MC2R* or *AR* overexpression in giant bilateral myelolipomas from poor-compliance CAH patients. Therefore, we can speculate that chronic ACTH and androgen stimulation may play a causative role in myelolipomas of poorly controlled CAH patients. In addition, X-chromosome inactivation pattern analysis revealed a polyclonal origin in all tumors, suggesting a stimulatory effect as a trigger for tumor development.

## Abbreviations

CAH: Congenital adrenal hyperplasia; ACTH: Adrenocorticotropic hormone; MC2R: Melanocortin 2 receptor; SF1: Steroidogenic factor 1; LEP: Leptin; AR: Androgen Receptor; ACTB: Beta-actin; GAPDH: Glyceraldehyde-3-phosphate dehydrogenase.

## Competing interest

The authors declared that they have no competing interest.

## Author contribution

MQA: data analysis, interpretation, drafting the manuscript. LCK: data acquisition, data analysis. LPB: data acquisition, data analysis, statistical analysis. AML: data acquisition, statistical analysis. BMP. Mariani: data acquisition, data analysis. MR: data acquisition, critical revision. OM: data acquisition, critical revision. FTD: performed the laparoscopic procedure of patients included in this study. BBM: critical revision, supervision. TASSB: conception and design, drafting the manuscript, critical revision, supervision. All authors read and approved the final manuscript.

## Pre-publication history

The pre-publication history for this paper can be accessed here:

http://www.biomedcentral.com/1472-6823/14/42/prepub
